# The association of competitiveness and sociodemographics with imposter phenomenon in a cohort of sport science college undergraduates

**DOI:** 10.1371/journal.pone.0346391

**Published:** 2026-06-23

**Authors:** Zachary Yukio Kerr, Ethan P. Sgarlata, Justin D. Dennis, Jeffrey A. Turner, Aliza K. Nedimyer, Jimikaye B. Courtney

**Affiliations:** 1 Department of Exercise and Sport Science, University of North Carolina at Chapel Hill, Chapel Hill, North Carolina, United States of America; 2 Human Movement Science Curriculum, University of North Carolina at Chapel Hill, Chapel Hill, North Carolina, United States of America; 3 STRONG Lab, Air Force Research Laboratory, Wright-Patterson Air Force Base, Fairborn, Ohio, United States of America; Alexandru Ioan Cuza University: Universitatea Alexandru Ioan Cuza, ROMANIA

## Abstract

**Introduction:**

Imposter phenomenon is where individuals experience continued feelings of self-doubt, with fears of being exposed as frauds. The intersectionality of different identities related to imposter phenomenon has rarely been examined in sport science student populations. This study examines the prevalence of imposter phenomenon among sport science undergraduate students and explores differences between different sociodemographic groups.

**Methods:**

This secondary analysis study examines 289 undergraduate students in a sport science course at a United States Southeastern university from Spring 2023 to Spring 2025. The Clance Imposter Phenomenon Scale (CIPS) measured imposter phenomenon, and the Revised Competitiveness Index (CI-R) examined competitiveness. Multiple linear regression examined associations of imposter phenomenon with sociodemographic variables and competitiveness subscales (enjoyment of competition, contentiousness).

**Results:**

Overall, 52.6% of students reported frequent or intense imposter characteristics (CIPS mean±SD = 62.1 ± 12.9). In the final model (*adjusted r² =* 26.7%), significant predictors included: being a senior versus first-year/sophomore (*b* = 5.6; 95%CI: 2.1, 9.2); and higher contentiousness (10%-increase-*b* = −1.5; 95%CI: −2.0, −0.9). Interaction terms included gender identity × first generation student status (p = 0.02) and varsity athlete × enjoyment of competition (p = 0.06). Among first generation students, those not identifying as men had higher imposter phenomenon than men (*b* = 7.4; 95%CI: 4.2, 10.6); among non-first generation students, no differences were found (*b* = −1.3; 95%CI: −7.9, 5.2). Among varsity athletes, for every 10% increase in the enjoyment of competition subscale, imposter phenomenon scores decreased by 2.1 (95%CI: −4.4, 0.1); among non-varsity athletes, enjoyment of competition was not associated with imposter phenomenon scores (10%-increase-*b* = 0.1; 95%CI: −0.7, 0.8).

**Discussion:**

Over half of the sample noted frequent and intense feelings of imposter phenomenon. Helping students consider their capabilities and potential for future success, despite academic setbacks, should be considered in resources to mitigate imposter phenomenon. Resources throughout a student's college tenure can help address imposter phenomenon at various stages of academic and professional development.

## Introduction

Imposter phenomenon (also colloquially referred to as imposter syndrome) is characterized as a psychological experience where individuals experience continued feelings of self-doubt, with innate fears of being exposed as a fraud, despite objective evidence of competence or success [1]. Imposter phenomenon was first examined in high-ranking, successful women in the business industry [1], but has since expanded to other professionals, alongside a focus on college students at both the undergraduate and graduate levels [2–13]. Additionally, imposter phenomenon is associated with psychological outcomes, including depression, anxiety, and stress, in student populations [5–7,14,15]. Beyond psychological outcomes, reduced academic performance and lower student satisfaction with their academic experiences has been associated with imposter phenomenon [2,16]. As a result, it is important to understand the prevalence of imposter phenomenon in student populations to help both students and their universities identify and develop strategies to mitigate imposter phenomenon and related maladaptive psychological and academic outcomes.

Recent scoping reviews of intervention studies note that there are promising approaches (including individual-, peer-, and institutional-based strategies) to mitigating imposter phenomenon [17,18], although most studies have focused on healthcare settings. These reviews also note that individual characteristics may play a critical role in creating targeted programs [17,18]. Within college students, women and underrepresented minority students experience heightened levels of imposter phenomenon [6,10,11,13,19–21]. Additional identities also may be associated with a student’s perception of feeling marginalized or inferior [22]. This could include being a first-generation student [3,23] or a varsity athlete. First generation students may face disadvantages in navigating college due to their lack of familial college experience [3]. Athletes may struggle with the dual identity they have as students and athletes, particularly balancing both roles together [24]. At the same time, individuals can have multiple marginalized identities simultaneously. For example, being both a woman and an underrepresented minority student may create unique conditions that further exacerbate experiences of imposter phenomenon. Intersectional and identity-linked imposter experiences have been examined in prior qualitative/mixed-methods and quantitative studies, with findings suggesting that imposter feelings often vary within demographic groups depending on how identities such as gender, racial or cultural identity, and educational background intersect with contextual stressors (e.g., belonging uncertainty, legitimacy concerns, or minority status stress) rather than being driven by any single identity alone [14,15,25–28]. However, comparatively few studies have explicitly modeled how intersecting identities jointly relate to imposter phenomenon in undergraduate student populations.

Along with individual characteristics, institutional contexts and their embedded cultures may be associated with imposter phenomenon [10,22,29]. For example, competitive academic environments, men-dominated STEM fields, or institutions with high-achieving reputations may exacerbate imposter phenomenon among individuals who are already vulnerable [21,30]. In particular, minority students may face stereotype threat (i.e., the fear of confirming a negative stereotype about their social group) and feelings of not belonging in academic environments where they are underrepresented. Beyond the structural characteristics of academic environments, the perceived competitiveness within these settings may also influence imposter phenomenon. Highly competitive environments can intensify self-doubt, particularly among students who already experience marginalization [31].

It is feasible to investigate how these factors are associated with imposter phenomenon in a university department that houses many first-generation students and students participating in varsity athletics. Sport science departments typically include a comprehensive scope of courses inclusive of exercise physiology, sport psychology, anatomy, biomechanics, kinesiology, and sport management. Sport science also functions as a feeder major into highly competitive professional and graduate programs in medicine or allied health fields (e.g., medical, nursing, physician assistant, physical therapy, occupational therapy) as well as research-oriented health sciences and professional sports. Examining sport science undergraduates therefore provides a valuable opportunity to understand how imposter phenomenon may emerge earlier in professional development, prior to formalized admissions gatekeeping and clinical training. Unlike medical students who typically undergo competitive admissions processes [10], sport science students may not face such application barriers, potentially providing insights into imposter phenomenon that are not solely driven by selective admissions processes. Other studies have examined undergraduate students in different disciplines (e.g., nursing, computer science) that often have more competitive admissions processes into their majors [2–9,12,13]. Additionally, sport science students may have to balance dual identities as both students and athletes [24], creating unique stressors that differ from traditional student populations. Together, these features position sport science as a distinct early training context for examining imposter phenomenon among students pursuing rigorous and competitive career tracks.

Research should specifically examine how student identities and competitive environments concurrently contribute to imposter phenomenon experiences. Understanding the prevalence and correlates of imposter phenomenon is critical for identifying which students are most likely to benefit from evidence-based interventions that can improve both psychological well-being and academic outcomes in diverse student populations. Coupled with the lack of research specific to undergraduate students in the sport science discipline, this study examines secondary data to address the following research questions:

**RQ1:** What is the prevalence of imposter phenomenon overall and among different groups of sport science undergraduate students?

**RQ2:** What factors are associated with imposter phenomenon in sport science undergraduate students?

**RQ2a:** Do sociodemographic factors interact with each other in their associations with imposter phenomenon?

**RQ2b:** Does the association between imposter phenomenon and competitiveness vary between different sociodemographic groups?

## Materials and methods

The methodology and measures of interest are detailed below. This study is a secondary analysis of data previously collected for educational purposes. The data are considered secondary because they were originally collected with the primary purpose of supporting student learning objectives in a research methods course. The specific research questions and analyses presented in this manuscript were developed after the original data collection was completed. This study was reviewed by the Institutional Review Board at the <<REDACTED>> and was deemed exempt from federal regulations. Data were fully anonymized in the original data collection and the study team did not have access to identifiers. As this was secondary data analysis, the requirement for informed consent was waived, although students were still informed about the potential uses of the data for future research purposes.

### Original purpose of dataset

These data were used for educational purposes for a research methods course for undergraduates in a sport science department at a university in the southeastern United States. As part of the university’s general curriculum in the College of Arts and Sciences, where this department is housed, students are required to take at least one “Research and Discovery” (R&D) course. The R&D courses focus on having students conduct novel research that gives them opportunities to apply data collection skills, consider strengths and limitations of the produced data, and reflect throughout the process.

This course in the sport science department is taught by multiple instructors who provide their specific sections with a unique topic to explore based on their area of expertise. The dataset in this study includes data from the nine sections taught by the lead author (ZYK) during the Spring 2023 to Spring 2025 semesters. Although this course was in a sport science department, the instructor opted to focus on a topic that they felt was relevant to all student experiences and thus allowed the application of R&D content to be relatable to students. Thus, the research project focused on student experiences with imposter phenomenon, with students completing a quantitative survey that included items about imposter phenomenon and competitiveness that was then used to understand measurement and applied statistics.

### Data collection

Enrolled students in the nine sections were asked to complete a Qualtrics survey developed by the instructor (ZYK) that included information on imposter phenomenon, competitiveness, and sociodemographics. Prior to implementation in the earliest section, the survey was pilot tested by four sport science undergraduate students. Feedback was then embedded.

During the middle of the semester, students completed the survey by scanning a QR code displayed on the classroom’s main screen. Those who were absent completed it outside of class time. Before administering the survey, the instructor explained its purpose both for educational use in the course and potential future research use. Students were informed that participation was voluntary and would not affect their course grade. By completing the survey, students provided implied consent for their anonymized data to be used for both educational and research purposes. To support privacy, the instructor left the room during completion and noted within the survey that responses would remain anonymous and not personally identifiable. The instructor thus could not identify individual participants during or after data collection. Further, the authors of the current study did not have access to information that could identify individual participants. The survey focused on general metrics rather than sensitive or detailed experiences, making it appropriate for classroom use.

For this study, all raw data were pulled at once from Qualtrics (on May 10, 2025), put into a new dataset, and manually checked to identify cases that were initially used for internal pilot testing (identified based on completion date) and incomplete data. Of the 304 data points, 15 cases were removed due to not completing the survey, resulting in a final dataset used for this analysis consisting of 289 students.

### Measures of interest

#### Imposter phenomenon.

The Clance Imposter Phenomenon Scale (CIPS) is a previously validated scale that examines one’s perception that their past success came from luck versus ability, often leading them to dismiss their own strengths [32]. It includes 20 five-point scale items ranging from “Not at all true” to “Very true,” with the overall score ranging from 20 to 100 (α = 0.90); higher scores suggest a greater presence of imposter phenomenon.

### Competitiveness

The Revised Competitiveness Index (CI-R) is a previously validated competitiveness index that examined one’s inclination to want to win or do better than others in interpersonal situations [33]. Competitiveness can manifest at institutional and systemic levels, with such environments often proliferating through interpersonal dynamics and individual behaviors. The CI‑R captures these individual‑level perceptions, which may reflect how broader competitive cultures are experienced by students [33]. The CI-R includes 14 five-point scale items ranging from “Strongly Disagree” to “Strongly Agree,” with the overall score ranging from 14 to 70 after accounting for reverse-coded items (α = 0.92); higher scores suggest a greater presence of competitiveness. The CI-R is comprised of two subscales: Enjoyment of competition (potential range of 9–45; α = 0.92) and Contentiousness (i.e., willingness to engage in confrontation and assert themselves in competitive situation; potential range of 5–25; α = 0.85).

### Sociodemographics

Participant characteristics were also collected as related to demographics and student enrollment. The six sociodemographics included: gender identity (Man, Woman, other); race/ethnicity (check-all-that-apply for White/Caucasian, Black/African-American, Latino/Hispanic, Asian/Asian-American/Pacific Islander, Indigenous, other); first generation student status (Yes, No); year in school [First year, Sophomore, Junior, Senior (including 5^th^ year), other]; varsity athlete status (Yes, No); and the degree track within this sample department’s major (General track, Fitness Professional, Sport Administration, other). A fill-in option was provided when “other” was chosen for gender identity, race/ethnicity, and sport science degree track.

### Statistical analyses

All analyses were conducted on SAS v9.4 (SAS Institute, Cary, NC). Frequencies of sociodemographic variables were computed. Frequencies and descriptives were also computed overall and per item on CI-R and CIPS. Means ± standard deviations (SD) of CIPS scores were calculated.

Frequencies for CI-R and CIPS were based on previously established categorizations [33]. For CI-R, this included a median split for the ranges of the overall scale (≥42 versus <42) and the subscales (Enjoyment of competition: ≥ 27 versus <27; Contentiousness: ≥ 15 versus <15) [33]. For CIPS, the presence of imposter characteristics was categorized into: few (≤40); moderate (41–60); frequent (61–80); and intense (>80) [32]. We presented variables in categorical form to improve interpretability and facilitate descriptive comparisons to prior studies. For regression analyses, however, we retained the quantitative nature of the scales to preserve information and statistical power, consistent with rigorous statistical practice  [34].

To examine **RQ1 (i.e., prevalence of imposter phenomenon)**, we calculated the prevalence of students with frequent and intense imposter characteristics overall and by sociodemographics. Categories were further grouped together to account for small cell counts. Gender identity merged “Woman” and other gender identities provided through the “fill-in” response into “Woman and nonbinary.” Because only two participants identified as nonbinary, pooling them with women both protected confidentiality and aligned with our aim to group individuals marginalized by gender identity. Race/ethnicity was categorized into clusters based on commonly found frequencies within the data: “White/non-Hispanic;” “Black/African American;” “White/Hispanic;” “Asian/Pacific Islander;” and “Mixed-race/Other.” First generation status and varsity athlete status categorizations were retained as “Yes” and “No.” Year in school merged “First-year” and “Sophomore” due to the low sample size of first-year students. Last, sport science degree track merged “Fitness Professional” with “General Track” due to the low sample size of Fitness Professional track students; all fill-in option noted not being in the sport science major and thus were coded as “Non-sport science major.”

We also calculated prevalences by various cross-sections of sociodemographics that were selected a priori: gender identity, varsity athlete status, first generation student status, and year in school. We did not calculate statistics for cross-sections of sociodemographics with race/ethnicity due to small cell counts and our reluctance to group together different racial/ethnic groups.

To examine **RQ2 (i.e., factors associated with imposter phenomenon),** we first computed a main effects model by running a multiple linear regression with CIPS (i.e., imposter phenomenon) as the outcome, and the six sociodemographic variables and the CI-R subscales (i.e., Enjoyment of competition and Contentiousness) as predictors. In addition to these variables, data collection academic year (2022/23, 2023/24, 2024/25) was included as a covariate; summer session classes were included in the previous academic year (e.g., Summer Session in 2024 was coded as “2023/24”).

For **RQ2a and RQ2b (i.e., presence of potential interaction terms),** it should be noted that these interaction analyses were exploratory in nature, and no *a priori* power calculations were conducted specifically for detecting interaction effects. As such, interaction findings should be interpreted with caution. To conduct these analyses, we computed an interaction terms model. We considered a model-building process to ensure good model fit while accounting for concerns about small cell sizes in cross-sections. First, we chose to consider the sociodemographics whose cross-sections were previously explored in RQ1: gender identity, varsity athlete status, first generation student status, and year in school. Second, we opted to consider each competitiveness subscale separately. Third, we considered only two-way interactions (i.e., no three-way interactions due to sample size concerns). Fourth, multiple linear regression models were fitted to assess each interaction term alongside its corresponding main effects. Interaction terms with p-values <0.10 were considered for further evaluation. Starting from the main effects model, separate models were subsequently constructed by adding one interaction term at a time, and their significance was assessed. Among these models, the interaction term that yielded the best model fit (i.e., the lowest AIC score and a Likelihood Ratio Test p-value < 0.10) was retained.

This process was repeated, with the continued examination of the addition of one of the remaining interaction terms to the updated model. This approach ensured that interaction terms were not added cumulatively in a fixed order but rather selected based on their individual contribution to improving model fit at each step. The process continued until no additional interaction terms improved model fit according to the predefined criteria. This step was repeated until no additional terms bettered model fit.

To probe multicollinearity for both models, variance inflation factors (VIF) were examined to ensure no model values were above the recommended cut-off point of 10 [35]. Assumptions underlying multiple linear regression (e.g., linearity, residual normality, homoscedasticity) were assessed and deemed acceptable for the reported models. Models incorporating transformed variables were explored to satisfy assumptions of linear regression. However, these transformations did not alter the direction or statistical significance of the effect estimates [i.e., unstandardized beta coefficients (*b*)]. Thus, effect estimates from models using untransformed variables were reported for ease of interpretation [34]. Effect estimates whose 95% confidence intervals (CI) excluded 0.0 were considered statistically significant.

The *adjusted r*^*2*^ for the main effects model and interaction terms model were calculated. Within each model, the partial r^2^ for each predictor variable in both models were also calculated. Partial r² values represent the unique proportion of variance explained by each predictor after controlling for all other variables. Larger partial r² values indicate predictors that contribute more uniquely to explaining variation in imposter phenomenon scores. In the interaction terms model, beta coefficients and partial r^2^ were not presented for the main effects included in interaction terms; further, the partial r^2^ for the interaction term was inclusive of these main effects.

## Results

Most participants identified as women (64.4%) and White/non-Hispanic (67.5%; Table 1). About one in six (17.6%) were first generation students and one in five (20.8%) were varsity athletes. Most were juniors and seniors (74.8%) and enrolled in the general sport science major track (74.0%).

**Table 1:** Characteristics of sample of undergraduate students in sport science research courses (n = 289).

Characteristic	n	%	Characteristic	n	%
Data collection academic year			Varsity athlete		
2022/23	65	22.5	Yes	60	20.8
2023/24	139	48.1	No	229	79.2
2024/25	85	29.4	**Sport science major track**		
**Gender identity**			Sport Administration	52	18.0
Men	101	34.9	General Track	214	74.0
Women	186	64.4	Fitness Professional	9	3.1
Nonbinary (fill-in)	2	0.7	Non-sport science major	14	4.8
**Race/ethnicity**			**Presence of imposter characteristics (CIPS)** ^**a**^		
White/non-Hispanic	195	67.5	Few (≤40)	15	5.2
Black/African American	27	9.3	Moderate (41–60)	122	42.2
White/Hispanic	24	8.3	Frequent (61–80)	128	44.3
Asian/Pacific Islander	19	6.6	Intense (>80)	24	8.3
Mixed-race/Other ^b^	24	8.3	**Competitiveness (CI-R)** ^**a**^		
**Year in school**			High (≥42)	222	76.8
Senior	93	32.2	Low (<42)	67	23.2
Junior	123	42.6	***Enjoyment of competition*** ^**a**^		
Sophomore	66	22.8	*High (≥27)*	*254*	*87.9*
First-year	7	2.4	*Low (<27)*	*35*	*12.1*
**First generation student**			***Contentiousness*** ^**a**^		
Yes	51	17.6	*High (≥15)*	*111*	*38.4*
No	238	82.4	*Low (<15)*	*178*	*61.6*

^a^Mean ± standard deviation (SD) scores for scales are as follows:

- Clance Imposter Phenomenon Scale (CIPS): 62.1 ± 12.9 (domain range of 20–100)

- Revised Competitiveness Index (CI-R): 48.5 ± 9.7 (domain range of 14–70)

- RCI Enjoyment of competition subscale: 35.6 ± 7.3 (domain range of 9–45)

- RCI Contentiousness subscale: 12.9 ± 4.7 (domain range of 5–25)

^b^The most common race/ethnicity in the “Mixed-race/Other” category was: White & Asian/Pacific Islander & non-Hispanic (n = 9). All other groupings had ≤ 3 participants.

Overall, 76.8% of the sample scored ≥42 on the CI-R (Table 1; mean±SD = 48.5 ± 9.7; S1 Table). The Enjoyment of competition subscale mean±SD was 35.6 ± 7.3 and the Contentiousness subscale mean±SD was 12.9 ± 4.7. The mean±SD of the CIPS was 62.1 ± 12.9 (S2 Table).

### RQ1: Prevalence of imposter phenomenon

Overall, 52.6% of the sample noted having frequent or intense imposter characteristics (i.e., CIPS scores >60) (Table 1). Among the sociodemographics, the categories with the highest average CIPS scores were: Asian/Pacific Islander (66.5 ± 14.6); first generation student (65.4 ± 14.0); not identifying as men (65.3 ± 11.7); and Mixed-race/Other (65.2 ± 12.9) (Table 2). Among cross-sections of sociodemographics explored, the groups with the highest average CIPS scores were: Seniors and first generation students (71.8 ± 14.5); First generation and non-varsity athletes (67.5 ± 12.1); and Seniors not identifying as men (67.3 ± 12.8). Within each of these categories and cross-sections, over 60% reported frequent or intense imposter characteristics.

**Table 2:** Imposter phenomenon scores among sample of undergraduate students in sport science research courses (n = 289).

Characteristic	n	CIPS score Mean ± SD	% with CIPS score >60 ^a^	Characteristic	n	CIPS score Mean ± SD	% with CIPS score >60 ^a^
**Gender identity**				**Gender identity, First generation student**			
Men	101	56.2 ± 13.0	29.7	Men, No	84	54.6 ± 11.5	23.8
Women and nonbinary ^b^	188	65.3 ± 11.7	64.9	Men, Yes	17	64.0 ± 17.0	58.8
**Race/ethnicity** ^**c**^				Women and nonbinary, No	154	65.1 ± 11.6	65.6
White/non-Hispanic	195	62.1 ± 12.4	52.3	Women and nonbinary, Yes	34	66.1 ± 12.6	61.8
Black/African American	27	56.3 ± 12.1	29.6	**Gender identity, Varsity athlete**			
White/Hispanic	24	62.0 ± 15.2	58.3	Men, No	69	58.8 ± 12.0	34.8
Asian/Pacific Islander	19	66.5 ± 14.6	63.2	Men, Yes	32	50.5 ± 13.2	18.8
Mixed-race/Other	24	65.2 ± 12.9	66.7	Women and nonbinary, No	160	65.8 ± 11.4	67.5
**Year in school**				Women and nonbinary, Yes	28	62.0 ± 13.4	50.0
1^st^ year/Sophomore	73	58.5 ± 12.1	42.5	**Year in school, First generation student**			
Junior	123	62.1 ± 12.7	55.3	1^st^ year/Soph., No	58	57.8 ± 12.0	41.4
Senior	93	64.9 ± 13.2	57.0	1^st^ year/Soph., Yes	15	61.3 ± 12.5	46.7
**First generation student**				Junior, No	105	62.0 ± 12.7	54.3
No	238	61.4 ± 12.6	50.8	Junior, Yes	18	62.4 ± 13.2	61.1
Yes	51	65.4 ± 14.0	60.8	Senior, No	75	63.3 ± 12.4	53.3
**Varsity athlete**				Senior, Yes	18	71.8 ± 14.5	72.2
No	229	63.7 ± 12.0	57.6	**Year in school, Varsity athlete**			
Yes	60	55.9 ± 14.4	33.3	1^st^ year/Soph., No	43	60.6 ± 10.6	46.5
**Sport science major track**				1^st^ year/Soph., Yes	30	55.5 ± 13.5	36.7
General track or FP	223	63.7 ± 12.8	58.3	Junior, No	101	63.5 ± 12.0	61.4
Sport Administration	52	56.6 ± 11.7	32.7	Junior, Yes	22	55.6 ± 14.3	27.3
Non-sport science major	14	57.2 ± 12.7	35.7	Senior, No	85	65.6 ± 12.4	58.8
**Gender identity, Year in school**				Senior, Yes	8	58.3 ± 19.3	37.5
Men, 1^st^ year/Soph.	30	51.3 ± 9.7	20.0	**First generation student, Varsity athlete**			
Men, Junior	34	54.9 ± 13.5	32.4	No, No	188	62.9 ± 11.8	55.3
Men, Senior	37	61.4 ± 13.2	35.1	No, Yes	50	55.7 ± 13.7	34.0
Women and nonbinary, 1^st^ year/Soph.	43	63.5 ± 11.1	58.1	Yes, No	41	67.5 ± 12.1	68.3
Women and nonbinary, Junior	89	64.9 ± 11.3	64.0	Yes, Yes	10	56.6 ± 18.3	30.0
Women and nonbinary, Senior	56	67.3 ± 12.8	71.4				

NOTE: CIPS = Clance Imposter Phenomenon Scale; FP = Fitness professional

^a^CIPS scores >60 indicate frequent or intense imposter characteristics

^b^The “women and nonbinary” gender identity group includes two students who self-identified as “nonbinary”

^c^Cross-sections with race/ethnicity and sport science major track were not conducted due to our hesitancy to further group different categories beyond what is presented

### RQ2: Factors associated with imposter phenomenon

In the main effects model (*adjusted r*^*2*^ = 25.0%), significant variables included gender identity (*partial r*^*2*^ = 5.1%), first generation student status (*partial r*^*2*^ = 2.8%), year in school (*partial r*^*2*^ = 3.7%), and contentiousness (*partial r*^*2*^ = 9.2%; Table 3). Participants not identifying as men had higher imposter phenomenon (*b* = 6.0; 95%CI: 2.9, 9.1) than men. First generation students had higher imposter phenomenon (*b* = 5.2; 95%CI: 1.5, 8.9) than non-first generation students. Seniors had higher imposter phenomenon than first-year/sophomore students (*b* = 5.6; 95%CI: 1.9, 9.2). And for every 10% increase in the contentiousness subscale, imposter phenomenon scores decreased by 1.6 (95%CI: −2.1, −1.0), corresponding with a 2% decrease on the CIPS scale.

**Table 3:** Multivariable linear regression models predicting imposter phenomenon scores among sample of undergraduate students in sport science research courses (n = 289).

Characteristic	Main effects model		Interaction terms model	
	Beta coefficient (*b*) (95%CI)	Partial r^2^ (%) ^a^	Beta coefficient (*b*) (95%CI)	Partial r^2^ (%) ^a^
**Data collection academic year**		0.6		0.9
2022-2023 (ref.)	0.		0.	
2023-2024	−1.8 (−5.3, 1.7)		−2.2 (−5.6, 1.1)	
2024-2025	−0.1 (−3.8, 3.6)		−0.2 (−3.7, 3.3)	
**Gender identity**		5.1		--
Men (ref.)	0.		--	
Women and nonbinary ^b^	6.0 (2.9, 9.1)*		--	
**Race/ethnicity**		2.5		2.4
White/non-Hispanic (ref.)	0.		0.	
Black/African American	−3.2 (−7.9, 1.6)		−3.1 (−7.6, 1.4)	
White/Hispanic	−2.5 (−7.7, 2.6)		−2.5 (−7.5, 2.4)	
Asian/Pacific Islander	1.5 (−4.1, 7.0)		2 (−3.3, 7.3)	
Mixed-race/Other	4.5 (−0.5, 9.4)		4 (−0.7, 8.7)	
**Year in school**		3.7		4.0
1^st^ year/Soph. (ref.) ^c^	0.		0.	
Junior	1.2 (−2.2, 4.6)		1.1 (−2.2, 4.3)	
Senior	5.6 (1.9, 9.2)*		5.6 (2.1, 9.2)*	
**First generation student**		2.8		--
No (ref.)	0.		--	
Yes	5.2 (1.5, 8.9)*		--	
**Varsity athlete**		1.2		--
No (ref.)	0.		--	
Yes	−3.3 (−7.0, 0.3)		--	
**Sport science major track**		1.1		0.9
General track or FP (ref.) ^c^	0.		0.	
Sport Administration	−3.3 (−7.0, 0.5)		−3.0 (−6.5, 0.6)	
Non-sport science major	−1.2 (−7.5, 5.1)		0.0 (−6.0, 6.1)	
**Revised Competitiveness Index**				
*Enjoyment of competition*		<0.1		--
10% score increase	0.0 (−0.8, 0.7)		--	
*Contentiousness*		9.2		8.5
10% score increase	−1.6 (−2.1, −1.0)*		−1.5 (−2.0, −0.9)*	
**Gender identity** × **First generation student status**		--		9.6^d^
Men vs. Women and nonbinary in First generation students	--		7.4 (4.2, 10.6)*	
Men vs. Women and nonbinary in Non-first generation students	--		−1.3 (−7.9, 5.2)	
**Varsity athlete status** × **Enjoyment of competition subscale**		--		2.6^e^
10% score increase in Varsity athletes	--		−2.1 (−4.4, 0.1)	
10% score increase in Non-varsity athletes	--		0.1 (−0.7, 0.8)	

NOTE: CI = Confidence interval; FP = Fitness professional; * = 95%CI does not include null value of 0.0

^a^Adjusted r^2^ of main effects model = 25.0%. Adjusted r^2^ of interaction terms model = 26.8%.

^b^The “women and nonbinary” gender group includes two nonbinary students

^c^Referent groups included two categories due to low sample size of first-year students (n = 7) and fitness professional students (n = 9). Hence these categories were merged with other categories.

^d^Beta coefficients and partial r^2^ are not presented for gender identity and first generation student status as they are main effects for the included interaction term. The partial r^2^ for the interaction term is inclusive of these main effects.

^e^Beta coefficients and partial r^2^ are not presented for varsity athlete status and enjoyment of competition subscale as they are main effects for the included interaction term. The partial r^2^ for the interaction term is inclusive of these main effects.

### RQ2 and RQ2b: Presence of interaction terms

To build the interaction terms models, we first examined multiple linear regression models with interaction terms and their main effect variables being the sole predictors. The models with the interaction terms yielding p-values <0.10 were: Gender identity × First generation student (p = 0.03); Gender identity × Enjoyment of competition (p = 0.07); First generation student × Contentiousness (p = 0.04); Varsity athlete × Enjoyment of competition (p = 0.007); and Varsity athlete × Contentiousness (p = 0.08).

These five interaction terms were then considered for the forward model building process (S3 Table). In Step 1, the interaction term that best improved model fit (i.e., yielded the lowest AIC score with a Likelihood Ratio Test p-value <0.10) was Gender identity × First generation student status (change in AIC = 3.92; Likelihood Ratio Test χ² = 5.92, p = 0.01). In Step 2, the interaction term that best improved model fit was Varsity athlete × Enjoyment of competition (change in AIC = 1.57; Likelihood Ratio Test χ² = 3.57, p = 0.06). In Step 3, no other interaction terms were found to improve model fit.

In the final interaction terms model (*adjusted r*^*2*^ = 26.8%), the significant findings for year in school (*partial r*^*2*^ = 4.0%) and contentiousness (*partial r*^*2*^ = 8.5%) were maintained (Table 3). Adjusting for all other covariates, seniors had higher imposter phenomenon than first-year/sophomore students (*b* = 5.6; 95%CI: 2.1, 9.2). And for every 10% increase in the contentiousness subscale, imposter phenomenon scores decreased by 1.5 (95%CI: −2.0, −0.9), corresponding with a 2% decrease on the CIPS scale.

**Fig 1:**
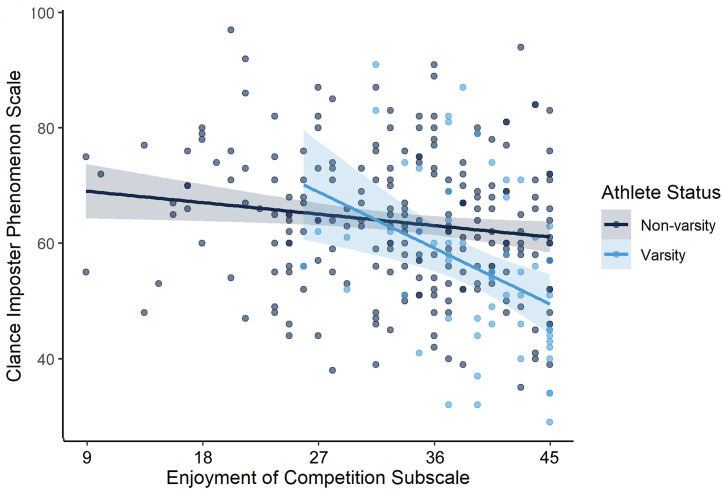
Scatterplots with lines-of-best-fit and 95% confidence interval bands examining association of enjoyment of competition and imposter phenomenon. In multivariable models, adjusting for all other covariates (inclusive of interaction terms), among varsity athletes, for every 10% increase in the Enjoyment of competition subscale, imposter phenomenon scores decreased by 2.1 (95%CI: −4.4, 0.1); among non-varsity athletes, no change in imposter phenomenon scores was found with changes in the Enjoyment of competition subscale (10% change *b* = 0.1; 95%CI: −0.7, 0.8).

Adjusting for all other covariates, the interaction term for Gender Identity × First generation student was statistically significant (p = 0.02). Among non-first generation students, there were no differences in imposter phenomenon by gender identity (*b* = −1.3; 95%CI: −7.9, 5.2); however, among first generation students, those identifying as women or nonbinary had higher imposter phenomenon than men (*b* = 7.4; 95%CI: 4.2, 10.6; Table 3). In fact, imposter phenomenon score was lower in non-first generation students identifying as men compared to all other groups (*b* for comparison to first generation students identifying as men = 10.7; 95%CI: 4.9, 16.6; *b* for comparison to non-first generation students not identifying as men = 9.4; 95%CI: 4.7, 14.1). Also, adjusting for all other covariates, the interaction term for Varsity athlete × Enjoyment of competition was within the 0.10 alpha threshold (p = 0.06). Among varsity athletes, for every 10% increase in the Enjoyment of competition subscale, imposter phenomenon scores decreased by 2.1 (95%CI: −4.4, 0.1), corresponding with a 2.6% decrease on the CIPS scale; among non-varsity athletes, enjoyment of competition was not associated with imposter phenomenon scores (10% change *b* = 0.1; 95%CI: −0.7, 0.8; Fig 1).

## Discussion

Understanding imposter phenomenon among college students is critical given its associations with reduced academic performance and satisfaction [2,16]. Previous research has also found the imposter phenomenon to be associated with broader indicators of student well‑being and psychological distress [5–7,14,15], emphasizing the need to understand how imposter experiences may differ across student demographic groups even when mental health outcomes were not directly assessed in this study. Students experiencing imposter phenomenon may struggle to accurately assess their capabilities and potential for future success, particularly when facing academic setbacks and failures. This misalignment between their self-perceptions and actual abilities can lead to missed opportunities due to underestimating their skills and result in diminished academic and future career trajectories. The current study examines a cohort of diverse students from the sport science discipline and allows for the exploration of how imposter phenomenon may manifest across the intersectionality of different identities. Within the sample, over half noted frequent and intense feelings of imposter phenomenon. Further, imposter phenomenon levels varied by different sociodemographic characteristics (e.g., gender identity, first generation student status, year in school) and levels of competitiveness. The findings highlight the need for developing and implementing evidence-based interventions about imposter phenomenon throughout a student’s tenure in college. This can include both formal and informal approaches, including educational programming throughout a student’s tenure, peer-led workshops, mentorship opportunities, and integration of content into advising or orientation sessions. Further, the findings can be situated within a broader literature linking imposter phenomenon to student well-being and identity‑related experiences, even as the specific patterns observed here differ across contexts and populations [14,15,25–28].

### General prevalence of imposter phenomenon

Similar to this sample, previous research examining college undergraduates found ranges of frequent and intense feelings of imposter phenomenon from 48.7% to 65.9% [9,10,12]. It is important to note that the sport science major from which this sample was recruited does not require an application process such as other major programs (e.g., computer science, business), which may mitigate feelings of rejection and subsequent imposter phenomenon. However, other characteristics of this sample may help explain this level of imposter phenomenon. This sample originates from a Research I institution that may have more rigorous coursework for students than other institutions. Students may also be affected by the institution’s perceived high academic prestige, particularly as it was recently qualified as a “New Ivy” [36]. Although some students have anecdotally expressed this perception, additional research is needed to empirically examine whether and how institutional reputation or broader academic climate meaningfully shape imposter phenomenon experiences.

Many students also apply to competitive graduate programs in physical therapy, occupational therapy, medical school, and sport administration. All these may result in greater feelings of imposter phenomenon due to students’ pressures to succeed and to compare their successes with one another. Future research should continue to examine the causal pathways and correlates of sociodemographics, imposter phenomenon, and academic outcomes while also identifying specific factors or situations that may create or inhibit feelings of imposter phenomenon. This combined approach may help to inform both the theoretical understanding of imposter phenomenon and the practical development of evidence-based interventions focused on aiding students’ progression through their college tenure.

### Factors associated with imposter phenomenon

Several sociodemographic characteristics were associated with imposter phenomenon, including gender identity, first generation student status, and year in school, which aligns with previous research noting the association between imposter phenomenon and various demographics and student characteristics [6,10,11,13,19,20,22,29]. Our findings also align with previous research suggesting that imposter phenomenon may not be uniformly experienced across broad demographic groups; imposter feelings can differ within gender or racial groups as a function of factors such as perceived belonging, academic legitimacy, and identity‑related stressors, rather than being driven by a single identity in isolation [14,15,28]. Although the present study did not observe strong associations between race and imposter phenomenon, prior research suggests that identity‑related experiences may shape imposter feelings in some student populations, warranting continued investigation across disciplinary and institutional contexts [14,15,28]. Overall, the imposter phenomenon may vary depending on how multiple identities intersect within particular educational contexts. The present findings reinforce the importance of examining these intersectional dynamics when seeking to understand imposter phenomenon among undergraduate students.

### Gender identity and first generation student status

Although previous research has noted the higher level of imposter phenomenon in women than men [6,10,11,13,19], our study replicated this only within non-first generation students. In fact, men who are non-first generation students may have less imposter phenomenon compared to everyone else, including first generation students of all gender identities. First generation students are acknowledged to face unique difficulties in college. For example, familial expectations may exist that place undue pressure on them to succeed; students’ families may also be unable to provide effective emotional or financial support as they are not familiar with the experience of being a college student [3]. First generation students may also lack role models that have gone to college. This limited support system and the potential lack of familial understanding and guidance could create heightened anxiety and uncertainty. Understanding the unique challenges of first generation students can help inform the development of targeted interventions, particularly as they transition into college.

This impact of being a first generation student appears to potentially erase the gender identity gap seen in non-first generation students, in which those not identifying as men had higher imposter phenomenon than those identifying as men. As previously considered [1], societal factors may contribute to this gap, including underrepresentation of women in certain academic and professional fields, and differences in perceptions of competence and achievement. With that said, we also highlight that two participants self-identified as “nonbinary” and were grouped with those identifying as “women” in the “women and nonbinary” category to ensure their inclusion in analyses. We acknowledge this limitation as it may not fully capture the unique experiences of non-cisgender individuals. A larger sample of non-cisgender individuals would be able to provide further insight in the role of gender identities that lie beyond the traditionalist dichotomy that exists.

### Year in school

Compared to first-year/sophomore students, seniors had higher imposter phenomenon. This contrasts previous findings in medical school populations which found that first-year medical students scored higher on the CIPS than final year medical students [10]. Several alternative explanations may account for this finding. First, seniors face transitions into either graduate programs or careers, creating acute uncertainty about their futures and external pressure to have secured post-graduation plans. The impending end of structured academic life may intensify self-doubt about whether they are truly prepared for the next stage. Second, the cumulative experience of four years in a competitive academic environment may have provided more opportunities to internalize perceived failures or to compare themselves unfavorably to peers, particularly through social media platforms focused on professional identities (e.g., LinkedIn). Third, seniors in sport science may face unique pressures as many apply to highly competitive graduate programs (e.g., medical school, physical therapy) where rejection rates are high, amplifying feelings of inadequacy. Although we do not have data on the proportion of seniors in our sample who already secured a job or admission to a graduate program, future research should examine how post-graduation security status moderates imposter phenomenon in seniors, and whether interventions timed to the senior year transition might be particularly beneficial. Thus, despite seniors having developed strategies to succeed in their coursework through time management and study habits, the findings highlight other areas of need that may have just as important implications on their mental and emotional health.

### Competitiveness

Previous research examining academic competitiveness found it to have no association with imposter phenomenon among college students [11]. However, our study used subscales of the CI-R, with each presenting different findings. First, those with higher levels of contentiousness (i.e., willingness to engage in confrontation and assert themselves in competitive situations) had lower levels of imposter phenomenon. Such students may be more apt to be resilient to criticism and are able to focus on their personal goals. Second, varsity athlete status moderated the association between enjoyment of competition and imposter phenomenon. Among varsity athletes, higher enjoyment of competition was associated with lower levels of imposter phenomenon; no association was found among non-varsity athletes. Prior experiences with lower levels of competition (e.g., high school, youth sports leagues) likely afforded these Division I athletes the opportunities to learn how to manage success and failures. As such, Division I athletes may enjoy the overall process of competition as opposed to viewing the potential failures that come with competition as threatening. This aligns with the stress appraisal literature [37], suggesting that Division I athletes may perceive the undergraduate academic environment as a challenge rather than a threat because their personal resources and abilities to cope exceed the demands placed on undergraduate students.

Only one study (a master thesis) has examined imposter phenomenon differences between athletes and non-athletes in college settings, finding that athletes had lower levels [38]. Thus, any skills that help them manage competition (e.g., managing expectations, building resilience from failure) in sports may align with the ability to counter insecurity, failure, and resultant imposter phenomenon as well [39]. Coaches may provide an additional source of support and have helped athletes learn how to approach constructive criticism and see it as an opportunity to improve [39]. In fact, a workplace intervention study found that one-on-one coaching was more effective in reducing imposter phenomenon than group-based training session [40]. Athletes may also have access to additional support that are typically unavailable to non-athletes (e.g., sports psychologists, academic tutors).

However, these interpretations should be balanced against potential selection biases. Division I athletes may enter college with pre-existing traits, such as higher confidence, self-efficacy, or resilience, that both facilitate athletic success and reduce vulnerability to imposter feelings, independent of their competitive experiences. Thus, lower imposter scores among athletes may reflect pre-existing psychological profiles rather than the developmental effects of sport participation. It is also possible that varsity athletes are answering items within the CIPS with varying reference points in that they are considering whether they feel like they are an imposter as a student, an athlete, or the dual roles they have as they balance both [24]. As this can also apply with other students in regard to other roles (e.g., employment outside of their education), future research should examine how students may vary in how they determine and to whom they reference their imposter phenomenon.

### Approaches to mitigate imposter phenomenon

A wide range of evidence-based interventions available throughout a student’s college tenure can help address imposter phenomenon at various stages of their academic and professional development. As noted in previous research [17,18], these interventions can and should occur at multiple levels of influence including individual- and peer-based (e.g., cognitive reflection and reframing, support groups) as well as institution-based (e.g., acknowledging the presence of imposter phenomenon, education). These can be incorporated into coursework [41] such as seminars designed to support first-year students as they transition into college, as well as programs that prepare seniors as they explore feelings associated with securing post-college employment, admission into graduate programs, or how they consider their past accomplishments. To maximize their impact, such interventions should incorporate strategies for building resilience, navigating challenges, and learning from failure.

With that said, acknowledging these strategies may be relevant to any identity group; thus, providing students with a “buffet” of resource options may help them to determine which strategies are more relevant and feasible to them. In addition, our interaction terms model only accounted for 26.8% of variance in imposter phenomenon, indicating that many other factors may have yet to be considered. R² values in the range of 0.10–0.30 are common and methodologically acceptable in social and behavioral research, where complex human experiences are influenced by numerous unmeasured factors [42]. Potential unmeasured factors include individual psychological characteristics (e.g., self-esteem, perfectionism, neuroticism, self-compassion) [11,19]; family and social support systems (e.g., parental education, family expectations, peer support networks) [3]; socioeconomic status and related financial stressors [31]; prior academic experiences and achievement histories; specific stressful life events or transitions; mental health conditions such as anxiety and depression that may both cause and result from imposter phenomenon [5–7,14,15]; institutional climate factors including perceptions of belonging, discrimination experiences, and mentorship availability [22,29]; and domain-specific factors such as perceived academic competitiveness beyond individual competitiveness traits [21,30,31]. Future studies with more comprehensive assessment of psychological, social, and institutional factors could better identify modifiable risk factors and inform more targeted intervention strategies.

### Limitations

As previously noted, these findings originate from a sample of college undergraduates from a Research I institution in the southeastern US that had enrolled in a research methods course in a sport science department. Findings may not be generalizable to those outside of this research class setting, department/major, university, and education level (e.g., graduate school, high school). For example, the proportion of students that are varsity athletes in our sample (20.8%) is likely higher than many other departments and may not be representative. In addition, because this is a single‑institution sample, institutional culture, regional factors, and department‑specific characteristics may uniquely shape students’ experiences of imposter phenomenon beyond what may be observed in other settings.

Caution should be taken with data interpretations due to information bias typical of survey study designs (e.g., social desirability bias). In addition, given the exploratory nature of our model‑building approach and the evaluation of multiple interaction terms, interpretation of the observed interaction effects should be done with caution, with emphasis on effect sizes and confidence intervals rather than statistical significance alone. Further, the data collection method originates from coursework that is required of undergraduates within this university, with the survey intake process being built into in-class activities. Although the instructor aimed to minimize potential bias and influence related to this data collection process, the mindset that a participant has when completing a survey as part of coursework versus opting in as part of a research study may have affected their responses. Because data were collected by the course instructor across semesters, subtle variations in how the survey was introduced or the instructor-student relationship may have introduced additional variability in responding. However, we aimed to keep data collection protocols consistent across each semester to mitigate these effects.

Also, while the models accounted for a reasonable amount of variance, missing variables could help explain additional variance in imposter phenomenon. For example, although understanding the impact of imposter phenomenon would have been improved with the inclusion of measures such as those related to academic performance and satisfaction [2,16], mental health conditions [5–7], socioeconomic status and related financial stressors [31], we believe that using student data in the classroom setting (the original intention of this dataset), even if they opted to share it, might be unethical and place students in uncomfortable positions. Therefore, these potentially important covariates were not collected and therefore could not be accounted for in our models. Likewise, as with any secondary data analysis study, caution should be taken with data interpretations as these analyses do not directly stem from the intended primary aims of the survey data collection, which was to help teach research methods to undergraduate students in a sport science course. Finally, the cross-sectional design limits causal inference. Observed associations may reflect pre-existing characteristics or unmeasured confounding rather than directional effects.

## Conclusion

Over half of the sample of college undergraduates noted frequent and intense feelings of imposter phenomenon. Findings also suggest that the intersectionality of identities (e.g., gender identity and first generation student status) may be associated with imposter phenomenon, which should be investigated in future studies. Helping students consider their capabilities and potential for future success, despite academic setbacks, should be considered in resources to mitigate imposter phenomenon. Resources throughout a student's college tenure can help address imposter phenomenon at various stages of academic and professional development.

## Supporting information

S1 Table Means and standard deviations for scales and individual items for Revised Competitiveness Index (α = 0.92). (PDF)

S2 Table Means and standard deviations for scales and individual items for Clance Imposter Phenomenon Scale (α = 0.90). (PDF)

S3 Table Model building for inclusion of interaction terms in multivariable linear regression model predicting imposter phenomenon scores. (PDF)

## References

[1] Clance PR, Imes SA. The imposter phenomenon in high achieving women: Dynamics and therapeutic intervention. Psychotherapy: Theory, Research & Practice. 1978;15(3):241–7. doi: 10.1037/h0086006

[2] Ménard AD, Chittle L. The impostor phenomenon in post‐secondary students: A review of the literature. Rev Educ. 2023;11(2):e3399.

[3] Holden CL, Wright LE, Herring AM, Sims PL. Imposter Syndrome Among First- and Continuing-Generation College Students: The Roles of Perfectionism and Stress. Journal of College Student Retention: Research, Theory & Practice. 2021;25(4):726–40. doi: 10.1177/15210251211019379

[4] Kuppusamy PDA, Heeranthy, Kangyan C, How LK, Htay MNN, Khobragade S, et al. How impostor syndrome affects academic performance and leadership virtues among undergraduate clinical year medical students. Asian J Med Health. 2022;20(10):172–80.

[5] Maftei A, Dumitriu A, Holman AC. They will discover I’m a fraud! The imposter syndrome among psychology students. Stud Psychol. 2021;63:337–51.

[6] Vilchez-Cornejo J, Romani L, Chávez-Bustamante S, Copaja-Corzo C, Sánchez-Vicente JC, Viera-Morón RD, et al. Imposter syndrome and its associated factors in medical students in six Peruvian faculties. Rev Colomb Psiquiatr (Engl Ed). 2023;52(2):113–20. doi: 10.1016/j.rcpeng.2021.04.006 37453817

[7] C. Quimbo J, Pearl C. Awa-ao K, Cyma S. Almendra F, R. Calonia D, E. Flores J. Qualitative Analysis of Academic Factors Contributing to Impostor Phenomenon Among University Undergraduates. IJRP. 2024;143(1). doi: 10.47119/ijrp1001431220246118

[8] McWilliams D, Block M, Hinson J, Kier KL. Impostor Phenomenon in Undergraduate and Doctor of Pharmacy Students at a Small Private University. Am J Pharm Educ. 2023;87(1):ajpe8728. doi: 10.5688/ajpe8728 34992067 PMC10159602

[9] Jacobs MD, Sasser JT. Impostor Phenomenon in Undergraduate Nursing Students: A Pilot Study of Prevalence and Patterns. J Nurs Educ. 2021;60(6):329–32. doi: 10.3928/01484834-20210520-05 34077317

[10] Franchi T, Russell-Sewell N. Medical Students and the Impostor Phenomenon: A Coexistence Precipitated and Perpetuated by the Educational Environment?. Med Sci Educ. 2022;33(1):27–38. doi: 10.1007/s40670-022-01675-x 37008445 PMC10060463

[11] Pákozdy C, Askew J, Dyer J. The imposter phenomenon and its relationship with self-efficacy, perfectionism and happiness in university students. Curr Psychol. 2024;43(6):5153–62.

[12] Lindsay J, Cropley S, Ramirez E. Prevalence of Impostor Phenomenon Among Final-Semester Baccalaureate Nursing Students. Dimens Crit Care Nurs. 2024;43(5):272–6. doi: 10.1097/DCC.0000000000000653 39074233

[13] Rosenstein A, Raghu A, Porter L. Identifying the Prevalence of the Impostor Phenomenon Among Computer Science Students. In: Proceedings of the 51st ACM Technical Symposium on Computer Science Education, 2020. 30–6. doi: 10.1145/3328778.3366815

[14] Cokley K, McClain S, Enciso A, Martinez M. An examination of the impact of minority status stress and impostor feelings on the mental health of diverse ethnic minority college students. J Multicult Couns Dev. 2013;41(2):82–95.

[15] Cokley KO, Bernard DL, Stone-Sabali S, Awad GH. Impostor Phenomenon in Racially/Ethnically Minoritized Groups: Current Knowledge and Future Directions. Annu Rev Clin Psychol. 2024;20(1):407–30. doi: 10.1146/annurev-clinpsy-081122-015724 38271635 PMC11245362

[16] El-Ashry AM, Taha SM, Elhay ESA, Hammad HA-H, Khedr MA, El-Sayed MM. Prevalence of imposter syndrome and its association with depression, stress, and anxiety among nursing students: a multi-center cross-sectional study. BMC Nurs. 2024;23(1):862. doi: 10.1186/s12912-024-02414-w 39605033 PMC11603883

[17] Siddiqui ZK, Church HR, Jayasuriya R, Boddice T, Tomlinson J. Educational interventions for imposter phenomenon in healthcare: a scoping review. BMC Med Educ. 2024;24(1):43. doi: 10.1186/s12909-023-04984-w 38191382 PMC10775670

[18] Para E, Dubreuil P, Miquelon P, Martin-Krumm C. Interventions addressing the impostor phenomenon: a scoping review. Front Psychol. 2024;15:1360540. doi: 10.3389/fpsyg.2024.1360540 38605843 PMC11007186

[19] Patzak A, Kollmayer M, Schober B. Buffering Impostor Feelings with Kindness: The Mediating Role of Self-compassion between Gender-Role Orientation and the Impostor Phenomenon. Front Psychol. 2017;8:1289. doi: 10.3389/fpsyg.2017.01289 28798714 PMC5526963

[20] Heslop G, Bonilla-Velez J, Faucett EA, Cabrera-Muffly C. Understanding and overcoming the psychological barriers to diversity: imposter syndrome and stereotype threat. Curr Otorhinolaryngol Rep. 2023;11(2):63–70.

[21] Collins KH, Price EF, Hanson L, Neaves D. Consequences of stereotype threat and imposter syndrome: The personal journey from stem-practitioner to stem-educator for four women of color. Taboo. 2020;19(4):10.

[22] Hewertson H, Tissa F. Intersectional Imposter Syndrome: How Imposterism Affects Marginalised Groups. The Palgrave Handbook of Imposter Syndrome in Higher Education. Springer International Publishing. 2022. p. 19–35. doi: 10.1007/978-3-030-86570-2_2

[23] Martin I. First generation African American college student-athletes and their lived experiences. Walden University. 2020.

[24] van Rens FECA, Ashley RA, Steele AR. Well-Being and Performance in Dual Careers: The Role of Academic and Athletic Identities. The Sport Psychologist. 2019;33(1):42–51. doi: 10.1123/tsp.2018-0026

[25] Chakraverty D. The Impostor Phenomenon Among Postdoctoral Trainees in STEM: A US-Based Mixed-Methods Study. IJDS. 2020;15:329–52. doi: 10.28945/4589

[26] Chakraverty D. A Cultural Impostor? Native American Experiences of Impostor Phenomenon in STEM. CBE Life Sci Educ. 2022;21(1):ar15. doi: 10.1187/cbe.21-08-0204 35225673 PMC9250367

[27] Cokley K, Smith L, Bernard D, Hurst A, Jackson S, Stone S, et al. Impostor feelings as a moderator and mediator of the relationship between perceived discrimination and mental health among racial/ethnic minority college students. J Couns Psychol. 2017;64(2):141–54. doi: 10.1037/cou0000198 28277731

[28] McClain S, Beasley ST, Jones B, Awosogba O, Jackson S, Cokley K. An examination of the impact of racial and ethnic identity, impostor feelings, and minority status stress on the mental health of Black college students. J Multicult Couns Dev. 2016;44(2):101–17.

[29] Rivera N, Feldman EA, Augustin DA, Caceres W, Gans HA, Blankenburg R. Do I Belong Here? Confronting Imposter Syndrome at an Individual, Peer, and Institutional Level in Health Professionals. MedEdPORTAL. 2021;17:11166. doi: 10.15766/mep_2374-8265.11166 34277932 PMC8257750

[30] Duncan L, Taasoobshirazi G, Vaudreuil A, Kota JS, Sneha S. An Evaluation of Impostor Phenomenon in Data Science Students. Int J Environ Res Public Health. 2023;20(5):4115. doi: 10.3390/ijerph20054115 36901122 PMC10001774

[31] London B, Anderson V, Downey G. Experiences of exclusion and marginalization: A study at the individual student level. In: Addison M, Breeze M, Taylor Y, editors. Diversity in American Higher Education: Toward a More Comprehensive Approach. Routledge. 2011. p. 195–206.

[32] Chrisman SM, Pieper WA, Clance PR, Holland CL, Glickauf-Hughes C. Validation of the Clance Imposter Phenomenon Scale. J Pers Assess. 1995;65(3):456–67. doi: 10.1207/s15327752jpa6503_6 16367709

[33] Houston JM, Harris PB, Patrick S. Revised Competitiveness Index (CI-R). International Handbook of Behavioral Health Assessment. Springer International Publishing. 2023. p. 1–20. doi: 10.1007/978-3-030-89738-3_30-1

[34] Harrell F. Regression modeling strategies: with applications to linear models, logistic and ordinal regression, and survival analysis. Springer. 2015.

[35] Hair JF, Anderson RE, Tatham RL, Black WC. Multivariate data analysis. Macmillan. 1995.

[36] Whitford E. The New Ivies 2025: 20 Great Colleges Employers Love. https://www.forbes.com/sites/emmawhitford/2025/03/26/the-new-ivies-2025-20-great-colleges-employers-love/ 2025. Accessed 2025 October 26.

[37] Blascovich J, Mendes WB. Challenge and threat appraisals: The role of affective cues. In: Forgas J, editor. Feeling and Thinking: The Role of Affect in Social Cognition. Cambridge University Press. 2000. p. 59–82.

[38] Swinney K. Comparing student-athletes and non-athletes on academic impostor syndrome. California State University, Northridge. 2020.

[39] Galli N, Gonzalez SP. Psychological resilience in sport: A review of the literature and implications for research and practice. International Journal of Sport and Exercise Psychology. 2014;13(3):243–57. doi: 10.1080/1612197x.2014.946947

[40] Zanchetta M, Junker S, Wolf A-M, Traut-Mattausch E. “Overcoming the Fear That Haunts Your Success” - The Effectiveness of Interventions for Reducing the Impostor Phenomenon. Front Psychol. 2020;11:405. doi: 10.3389/fpsyg.2020.00405 32499733 PMC7242655

[41] Cisco J. Using academic skill set interventions to reduce impostor phenomenon feelings in postgraduate students. J Furth High Educ. 2020;44(3):423–37.

[42] Ozili PK. The acceptable R-square in empirical modelling for social science research. Social research methodology and publishing results: A guide to non-native English speakers. IGI Global. 2023. p. 134–43.

