# Supplementary material for: The association of competitiveness and sociodemographics with imposter phenomenon in a cohort of sport science college undergraduates
Source: PLoS One. 2026 Jun 23;21(6):e0346391. doi: 10.1371/journal.pone.0346391 (PMC13289916; doi:10.1371/journal.pone.0346391)
Supplement: S1 Table — (PDF) [file pone.0346391.s001.pdf]

Supplemental Table 1. Means and standard deviations for scales and individual items for Revised Competitiveness Index (α=0.92)

Items	Mean ± SD	
**Total scale**	**48.5 ± 9.7**	
**Items**	**Mean ± SD**	**% responding "Strongly agree"**
**Enjoyment of competition subscale (α=0.92)**	**35.6 ± 7.3**	
2. I am a competitive individual.	4.4 ± 0.9	56.1
1. I like competition.	4.2 ± 1.0	48.4
3. I enjoy competing against an opponent.	4.1 ± 1.0	47.1
9. I often try to outperform others.	4.0 ± 1.0	36.3
5. I get satisfaction from competing with others.	3.9 ± 1.0	33.2
8. I try to avoid competing with others. [a]	3.7 ± 1.2	3.8
4. I don't like competing against other people. [a]	3.8 ± 1.1	3.1
6. I find competitive situations unpleasant. [a]	3.6 ± 1.1	2.8
7. I dread competing with others. [a]	3.8 ± 1.1	2.4
**Contentiousness subscale (α=0.85)**	**12.9 ± 4.7**	
10. I try to avoid arguments. [a]	2.2 ± 1.1	34.3
12. I often remain quiet rather than risk hurting another person. [a]	2.4 ± 1.2	26.6
11. I will do almost anything to avoid an argument. [a]	2.8 ± 1.3	22.1
14. In general, I go along with the group rather than create conflict. [a]	2.6 ± 1.1	17.3
13. I don't enjoy challenging others even when I think they are wrong. [a]	3.0 ± 1.2	13.5

NOTE: Higher scores suggest greater presence of competitiveness. Scale has a potential range of 14-70, with Enjoyment of competition and Contentiousness subscales having potential ranges of 9-45 and 5-25, respectively. Each item was measured on a 5-point scale: Strongly Disagree, Slightly Disagree, Neither Disagree nor Agree, Slightly Agree, Strongly Agree. Items for each subscale are ordered from highest to lowest mean score.
[a] Item was reverse-coded; presented mean reflects this reverse coding (i.e., higher scores suggest greater presence of competitiveness)
