# Supplementary material for: The association of competitiveness and sociodemographics with imposter phenomenon in a cohort of sport science college undergraduates
Source: PLoS One. 2026 Jun 23;21(6):e0346391. doi: 10.1371/journal.pone.0346391 (PMC13289916; doi:10.1371/journal.pone.0346391)
Supplement: S2 Table — (PDF) [file pone.0346391.s002.pdf]

Supplemental Table 2. Means and standard deviations for scales and individual items for Clance Imposter Phenomenon Scale (α=0.90)

Items	Mean ± SD	% responding "Often" or "Very true"
**Total scale**	**62.1 ± 12.9**	
17. I often compare my ability to those around me and think they may be more intelligent than I am.	3.7 ± 1.1	59.5
7. I tend to remember the incidents in which I have not done my best more than those times I have done my best.	3.6 ± 1.1	57.4
1. I have often succeeded on a test or task even though I was afraid that I would not do well before I undertook the task.	3.6 ± 0.8	57.8
19. If I'm going to receive a promotion or gain recognition of some kind, I hesitate to tell others until it is an accomplished fact.	3.6 ± 1.1	56.1
18. I often worry about not succeeding with a project or examination, even though others around me have considerable confidence that I will do well.	3.5 ± 1.0	48.4
12. I'm disappointed at times in my present accomplishments and think I should have accomplished much more.	3.4 ± 1.1	49.5
2. I can give the impression that I'm more competent than I really am.	3.3 ± 0.9	37.4
20. I feel bad and discouraged if I'm not "the best" or at least "very special" in situations that involve achievement.	3.2 ± 1.2	39.8
14. I'm often afraid that I may fail at a new assignment or undertaking even though I generally do well at what I attempt.	3.2 ± 1.0	37.4
4. When people praise me for something I've accomplished, I'm afraid I won't be able to live up to their expectations of me in the future.	3.1 ± 1.2	36.3
10. It's hard for me to accept compliments or praise about my intelligence or accomplishments.	3.1 ± 1.2	37.0
16. If I receive a great deal of praise and recognition for something I've accomplished, I tend to discount the importance of what I've done.	3.1 ± 1.1	37.0
3. I avoid evaluations if possible and have a dread of others evaluating me.	2.9 ± 1.1	28.4
15. When I've succeeded at something and received recognition for my accomplishments, I have doubts that I can keep repeating that success.	2.9 ± 1.1	27.7
13. Sometimes I'm afraid others will discover how much knowledge or ability I really lack.	2.9 ± 1.1	31.5
6. I'm afraid people important to me may find out that I'm not as capable as they think I am.	2.9 ± 1.2	30.4
5. I sometimes think I obtained my present position or gained my present success because I happened to be in the right place at the right time or knew the right people.	2.6 ± 1.1	21.1
8. I rarely do a project or task as well as I'd like to do it.	2.6 ± 0.9	13.1
11. At times, I feel my success has been due to some kind of luck.	2.6 ± 1.1	20.1
9. Sometimes I feel or believe that my success in my life or in my job has been the result of some kind of error.	2.1 ± 1.0	10.0

NOTE: Higher scores suggest greater presence of imposter syndrome. Scale has a potential range of 20-100. Each item was measured on a 5-point scale: Not at all true, Rarely, Sometimes, Often, Very true. Items are ordered from highest to lowest mean score.
