# Supplementary material for: The association of competitiveness and sociodemographics with imposter phenomenon in a cohort of sport science college undergraduates
Source: PLoS One. 2026 Jun 23;21(6):e0346391. doi: 10.1371/journal.pone.0346391 (PMC13289916; doi:10.1371/journal.pone.0346391)
Supplement: S3 Table — (PDF) [file pone.0346391.s003.pdf]

Supplemental Table 3. Model building for inclusion of interaction terms in multivariable linear regression model predicting imposter phenomenon scores

		AIC		Likelihood ratio test		
**Step**	**Model**	**Score**	**Decrease from previous step**	**-2 Log Likelihood**	χ^2	**p-value**
0	**Main effects model** (see Table 3)	**2232.77**	**--**	**2198.77**	**--**	--
1	**Add Gender identity × First generation student status**	**2228.85**	**3.92**	**2192.85**	**5.92**	**0.01**
	Add Varsity athlete status × Enjoyment of competition subscale	2231.08	1.70	2195.08	3.70	0.05
	Add Varsity athlete status × Contentiousness subscale	2232.36	0.41	2196.36	2.41	0.12
	Add Gender identity × Enjoyment of competition subscale	2232.50	0.27	2196.50	2.27	0.13
	Add First generation student status × Contentiousness subscale	2232.75	0.02	2196.75	2.02	0.15
2	**Add Varsity athlete status × Enjoyment of competition subscale**	**2227.29**	**1.57**	**2189.29**	**3.57**	**0.06**
	Add Varsity athlete status × Contentiousness subscale	2228.58	0.28	2190.58	2.28	0.13
	Add Gender identity × Enjoyment of competition subscale	2228.99	-0.14	2190.99	1.86	0.17
	Add First generation student status × Contentiousness subscale	2229.30	-0.45	2191.30	1.55	0.21
3	Add First generation student status × Contentiousness subscale	2227.55	-0.26	2187.55	1.74	0.19
	Add Varsity athlete status × Contentiousness subscale	2228.29	-1.01	2188.29	0.99	0.32
	Add Gender identity × Enjoyment of competition subscale	2228.35	-1.06	2188.35	0.94	0.33

NOTE: Multiple linear regression models were run with the interaction terms and their main effect variables being the sole predictors. Those interaction terms yielding p-values <0.10 were considered for inclusion in the model building process. These include the interactions of: Gender identity and First generation student status (p=0.03); Gender identity and Enjoyment of competition subscale (p=0.07); First generation student status and Contentiousness subscale (p=0.04); Varsity athlete status and Enjoyment of competition subscale (p=0.007); and Varsity athlete status and Contentiousness subscale (p=0.08). In the forward model building process, we began with the full model including only main effects. We created separate models by adding one interaction term at a time. Among these models, the interaction term that yielded the best model fit (i.e., the lowest AIC score and a Likelihood Ratio Test p-value < 0.10) was retained. Above, the models are ordered within each step from lowest to highest AIC. This process was repeated, with the continued examination of the addition of one of the remaining interaction terms to the updated model. The process continued until no additional interaction terms improved model fit according to the predefined criteria. This step was repeated until no additional terms bettered model fit.
